# Microglia Activation in Retinal Ischemia Triggers Cytokine and Toll-Like Receptor Response

**DOI:** 10.1007/s12031-020-01674-w

**Published:** 2020-08-24

**Authors:** Natalie Wagner, Sabrina Reinehr, Marina Palmhof, David Schuschel, Teresa Tsai, Emely Sommer, Viktoria Frank, Gesa Stute, H. Burkhard Dick, Stephanie C. Joachim

**Affiliations:** Experimental Eye Research, University Eye Hospital, Ruhr-University Bochum, In der Schornau 23-25, 44892 Bochum, Germany

**Keywords:** Ischemia, Microglia, Cytokine, Toll-like-receptor, NFκB, MyD88, Caspase, IL-1β

## Abstract

**Abstract:**

Mechanisms and progression of ischemic injuries in the retina are still incompletely clarified. Therefore, the time course of microglia activation as well as resulting cytokine expression and downstream signaling were investigated. Ischemia was induced in one eye by transiently elevated intraocular pressure (60 min) followed by reperfusion; the other eye served as a control. Eyes were processed for RT-qPCR and immunohistochemistry analyses at 2, 6, 12, and 24 h as well as at 3 and 7 days. Already 2 h after ischemia, more microglia/macrophages were in an active state in the ischemia group. This was accompanied by an upregulation of pro-inflammatory cytokines, like IL-1β, IL-6, TNFα, and TGFβ. Activation of TLR3, TLR2, and the adaptor molecule Myd88 was also observed after 2 h. NFκB revealed a wave-like activation pattern. In addition, an extrinsic caspase pathway activation was noted at early time points, while enhanced numbers of cleaved caspase 3^+^ cells could be observed in ischemic retinae throughout the study. Retinal ischemia induced an early and strong microglia/macrophage response as well as cytokine and apoptotic activation processes. Moreover, in early and late ischemic damaging processes, TLR expression and downstream signaling were involved, suggesting an involvement in neuronal death in ischemic retinae.

**Graphical Abstract:**

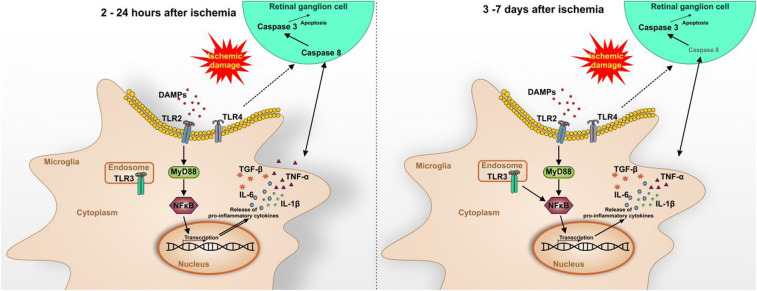

**Electronic supplementary material:**

The online version of this article (10.1007/s12031-020-01674-w) contains supplementary material, which is available to authorized users.

## Introduction

Ischemia is based on limited blood supply in a localized area, due to the blockage of blood vessels in the affected area. This in turn causes energy exhaustion and ultimately cell death. In the retina, ischemia develops as a result of capillary blockage, results in non-perfusion of this region, and leads to dysfunction as well as death of neuronal cells (Osborne et al. 2004). This is accompanied by glia cell activation followed by a secretion of cytotoxic mediators, including cytokines. Ischemic processes occur in several eye diseases, such as diabetic retinopathy or glaucoma (Schmidl et al. 2011; Sim et al. 2013; Terelak-Borys et al. 2012).

Glaucoma is the second most common cause of blindness worldwide, with a rising trend due to an aging society (EGS 2014; Quigley and Broman 2006). Furthermore, glaucoma not only leads to health problems but also results in economic damage. In Europe, the cost of treating glaucoma patients rose from 455 € to 969 € per person and, in the USA, the cost for treatment for this disease is estimated at $2.5 billion a year (Lazcano-Gomez et al. 2016; Traverso et al. 2005). In general, glaucoma is a multi-factorial disease characterized by a chronic loss of retinal ganglion cells (RGCs) and their axons (Casson et al. 2012; EGS 2014). Currently, the elevated intraocular pressure (IOP) is the only risk factor that can be handled by medical or surgical treatment. Unfortunately, in many cases, optic nerve and RGC degeneration as well as visual field loss continue on a long-term basis (Chang and Goldberg 2012; Pascale et al. 2012). Consequently, improved knowledge about the ischemia downstream processes can help to develop novel treatment options.

A common animal model to study pathological ischemic processes is the retinal ischemia/reperfusion model. Here, the IOP is temporarily increased by the infusion of fluid into the anterior chamber with a subsequent natural reperfusion. The consequence is a lower oxygenation capacity and supply of nutrients followed by the formation of oxidative stress during the recurring blood flow (Kaur et al. 2008; Kim et al. 2013; Minhas et al. 2012). It is known that RGCs and other cell types of the inner retinal layers are mainly affected in this model (Palmhof et al. 2019a; Schmid et al. 2014; Zheng et al. 2004). Previous studies on retinal ischemia have mostly analyzed only one or a few time points. Therefore, our research group recently investigated how cell types are affected over time in this model. At 3 days, a significant decrease in the total retinal thickness was observed, while changes in the RGC layer and a specific loss of RGCs were already noted after a few hours, which continued to increase over time (Palmhof et al. 2019a).

Microglia are the only permanent immune cells of the central nervous system (CNS) (Hanisch and Kettenmann 2007). In line, many molecules and conditions can trigger a transformation of resting or surveying microglia to alerted or reactive states. Therefore, they are the first line of defense against neuronal injury or ischemia (Lyons et al. 2000; Schmid et al. 2014). A few hours after ischemia, inflammation as well as an activation of glial cells and angiogenesis occurs. In consequence, proinflammatory cytokines, like interleukin (IL)-1β, IL-6, and γ-interferon, are activated (Berger et al. 2008; Rivera et al. 2013; Yoneda et al. 2001). In general, cytokines can affect not only the cells they are released from but also adjacent ones, which lead to critical misbalance in retinal diseases. Increased levels of IL-1 and other inflammatory markers were detected in vitreous samples of diabetic retinopathy patients (Reverter et al. 2009). Also, in retinal ischemia animal models, increased VEGF (Abcouwer et al. 2010; Chen et al. 2012; Hayashi et al. 1996) and IL-6 (Hangai et al. 1996; Sanchez et al. 2003; Wang et al. 2006) levels were observed after injury. IL-6 alterations are already detectable about 2 h after ischemia (Sanchez et al. 2003). A comparable early response occurs after ischemia/reperfusion brain injury (Berti et al. 2002). These effects could contribute to secondary cellular responses that lead to further retinal damage.

Toll-like receptors (TLRs) are pattern recognition receptors that play an important role in the initiation of the immune system. In line, TLRs generate signals which are passed through the NFκB signaling pathway as well as the MAP kinase pathway to recruit pro-inflammatory cytokines which in turn promote inflammatory response (Vidya et al. 2018). Interestingly, when microglia are triggered by a stimulus, such as oxidative injury, activated microglia express higher levels of TLRs in the CNS (Carpentier et al. 2008). Furthermore, previous studies showed a wide range of TLRs on microglia/macrophages which can be stimulated by secreted cytokines. This in turn reinforces the proinflammatory environment as well as neuronal dysfunction and thus enhances neuronal cell death (Chen et al. 2017; Li et al. 2014).

RGCs are strongly affected in the retinal ischemia-reperfusion model (Palmhof et al. 2019b). It is known that RGC cell death in this model is caused mainly by apoptosis (Lam et al. 1999; Selles-Navarro et al. 1996). Several initiator caspases (e.g. caspase 8 or 9) trigger both caspase pathways (intrinsic and extrinsic). Through effectors, like caspase 3, the common final track of the signaling cascade is activated (Erekat 2018; Kurokawa and Kornbluth 2009).

The aim of this project was to depict the timeline of microglia/macrophage response due to retinal ischemia. In addition, the importance of microglia associated cytokine expression as well as TLR signaling and apoptosis for retinal ischemia will be analyzed in detail. We could show that already 2 h after ischemia, microglia as well as their pro-inflammatory cytokines were enhanced. This was accompanied by TLR signaling and apoptosis.

The improved knowledge of the ischemia-related downstream processes and the importance of microglia gained from this study will then contribute to the development of new treatment options.

## Methods

### Ischemia/Reperfusion Model

Male brown Norway rats (7–8 weeks of age; Charles River Laboratories, Sulzfeld, Germany) were used for this ischemia/reperfusion project. The study was approved by the animal care committee of North Rhine-Westphalia (Germany); all experiments were carried out in accordance with the ARVO statement for the use of animals in ophthalmic and vision research. Rats were housed under environmentally controlled conditions (12-h light-dark cycle) with free access to chow and water.

**Fig. 1:**
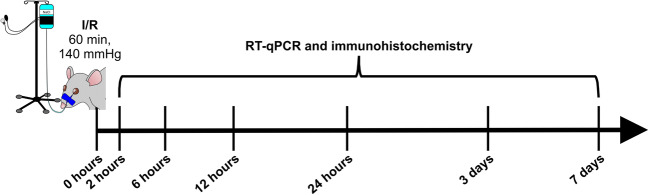
Study timeline. Ischemia was induced for 60 min by elevating the intraocular pressure to 140 mmHg in one eye per rat. The other eye remained untreated and served as control. Afterwards, the retinal tissue was analyzed 2, 6, 12, and 24 h as well as 3 and 7 days after ischemia by quantitative real-time PCR and immunohistochemistry. I/R ischemia/reperfusion, RT-qPCR quantitative real-time PCR

Retinal ischemia/reperfusion was induced as previously described (Joachim et al. 2017; Palmhof et al. 2018; Schmid et al. 2014). An elevated IOP of 140 mmHg was applied to the right eye of each animal for 60 min, followed by reperfusion (Fig. 1). The left, untreated eye served as a control.

Eyes were obtained 2, 6, 12, 24 h, and 3 as well as 7 days after ischemia induction (Fig. 1). The tissue was used for RT-qPCR analysis (*n* = 5/group) or immunohistochemistry (*n* = 7–8/group). For RT-qPCR, retinae were dissected and snap frozen in a lysis buffer (Sigma-Aldrich, Steinheim, Germany) in liquid nitrogen. For immunohistology, eyes were fixed in 4% paraformaldehyde, incubated in 30% sucrose, and embedded in optical cutting temperature medium (Tissue-Tek; Thermo Fisher Scientific, Cheshire, UK). Then, retinal cross sections were prepared (10 μm).

### Quantitative Real-time PCR Analysis

Retinae (*n* = 5/group/time point) were used for RNA preparation and cDNA synthesis as previously described (Osborne et al. 2004; Reinehr et al. 2019). A PikoReal 96 Real-time PCR System (Thermo Fisher Scientific) with SYBR Green (DyNAmo Flash SYBR Green qPCR Kit; Thermo Fisher Scientific) was used for RT-qPCR. Primer efficiencies of each primer set were calculated based on a dilution series of 5 to 125 ng cDNA. Ct values of the house-keeping genes *β-actin* (*Actb)* and *Cyclophilin* (*Ppid*) were applied for normalization and relative quantification of gene expressions (Table 1).

**Table 1:** List of primer pairs used for RT-qPCR analyses. For relative quantification of mRNA levels, the house-keeping genes were *β-actin* (*Actb)* and *Cyclophilin* (*Ppid*) . The primer sequence, the predicted amplicon size, and the primer efficiency are indicated. bp base pairs, F forward, R reverse

Gene	Primer sequence	Amplicon size (bp)	Primer efficiency
*Actb-F*	cccgcgagtacaaccttct	72	1.000
*Actb-R*	cgtcatccatggcgaact		
*Casp3*-F	ccgacttcctgtatgcttactcta	70	1.000
*Casp3*-R	catgacccgtcccttgaa		
*Casp8*-F	agagcctgagggaaagatgtc	72	1.000
*Casp8*-R	tcacatcatagttcacgccagt		
*Casp9*-F	cgtggtggtcatcctctctc	81	1.000
*Casp9*-R	gagcatccatctgtgccata		
*Cd68-F*	ctcacaaaaaggctgccact	60	1.000
*Cd68-R*	ttccggtggttgtaggtgtc		
*Iba1-F*	ctccgaggagacgttcagtt	96	0.855
*Iba1-R*	tttttctcctcatacatcagaatcatcagaat		
*Il1b-F*	tgtgatgaaagacggcacac	70	1.000
*Il1b-R*	cttcttctttgggtattgtttgg		
*Il6-F*	cctggagtttgtgaagaacaact	142	1.000
*Il6-R*	ggaagttggggtaggaagga		
*Myd88-F*	atgaactgaaggaccgcatc	127	1.000
*Myd88-R*	cccagttcctttgtctgtgg		
*Nfκb-F*	ctggcagctcttctcaaagc	70	0.960
*Nfκb-R*	ccaggtcatagagaggctcaa		
*Ppid-F*	tgctggaccaaacacaaatg	88	1.000
*Ppid-R*	cttcccaaagaccacatgct		
*Tmem119-F*	ttctggctgctactcagaacc	68	1.000
*Tmem119-R*	ttttgttccctccccactg		
*Tgfb-F*	cctggaaagggctcaacac	99	1.000
*Tgfb-R*	tgccgtacacagcagttctt		
*Tlr2-F*	tgctatgatgcctttgtttcc	60	1.000
*Tlr2-R*	catgaggttctccacccaat		
*Tlr3-F*	cttgtcatcaaatccacttaaagagt	70	1.000
*Tlr3-R*	gaggacgaataacttgccaatc		
*Tlr4-F*	ccttgagaaagtggagaagtcc	61	1.000
*Tlr4-R*	gctaagaaggcgatacaattcg		
*Tnfa-F*	gcccagaccctcacactc	99	1.000
*Tnfa-R*	ccactccagctgctcctct		

### Immunohistochemistry

Six retinal cross sections (*n* = 7–8/group/time point) were stained per immunohistochemistry marker (Table 2) (Palmhof et al. 2019a). Therefore, the sections were first thawed and then rehydrated in PBS, followed by blocking in 10–20% appropriate serum with or without 1% BSA in 0.1% Triton X-100 in PBS. Specific first and secondary antibodies were applied (Table 2), followed by DAPI (4′,6-diamidin-2-phenylindol; Serva Electrophoresis, Heidelberg, Germany) to visualize cell nuclei. For each staining, negative controls were included, with secondary antibodies only.

**Table 2:** List of used primary and secondary antibodies for immunohistochemistry, including cell type, dilution, and distributor

Primary antibody	Antigen	Dilution	Distributor	Secondary antibody	Dilution	Distributor
Rabbit anti-cleaved caspase 3	Cleaved caspase 3	1:100	Sigma-Aldrich	Donkey anti-rabbit Alexa 555	1:500	Invitrogen
Mouse anti-caspase 8	Caspase 8	1:100	Antibodies online	Donkey anti-mouse Alexa 488	1:500	Invitrogen
Mouse anti-ED1	Activated microglia	1:200	Millipore	Goat anti-mouse Alexa 488	1:500	Invitrogen
Rabbit anti-Iba1	Microglia	1:400	Wako	Goat anti-rabbit Cy 3	1:500	Linaris
Goat anti-IL-1β	Interleukin 1β	1:200	Millipore	Donkey anti-goat Alexa 488	1:500	Dianova
Mouse anti-NFκB	NFκB	1:500	Santa Cruz	Goat anti-mouse Alexa 488	1:600	Invitrogen
Rabbit anti-TLR3	Toll-like receptor 3	1:100	Santa Cruz	Donkey anti-rabbit Alexa 555	1:400	Invitrogen
Rabbit anti-TLR4	Toll-like receptor 4	1:400	Abcam	Donkey anti-rabbit Alexa 555	1:500	Invitrogen

Four pictures per section were taken with a fluorescence microscope (Axio Imager M1 and M2; Carl Zeiss Microscopy, Jena, Germany). All images were transferred to Corel Paint Shop Photo Pro (V 13; Corel Corporation, Fremont, CA, USA), masked, and equal excerpts were cut out.

Cleaved caspase 3^+^, caspase 8^+^, ED1^+^, Iba1^+^, IL-1β^+^, NFkB^+^, TLR3^+^, and TLR4^+^ cells were counted under masked conditions using ImageJ software (V 1.44p; NIH, Bethesda, MD, USA). In regard to ED1^+^ cells, only the ones colocalized with Iba1^+^ cells were included in the cell count.

### Statistical Analysis

RT-qPCR data are presented as median ± quartile + minimum/maximum and immunohistology data as mean ± standard error mean (SEM) with *p < 0.05, **p < 0.01, and ***p < 0.001. Relative expression variations in RT-qPCR analyses were done by REST© software (QIAGEN GmbH, Hilden, Germany) using a pairwise fixed reallocation and randomization test. Regarding immunohistochemistry, both groups were compared per point in time using Student’s *t* test (Statistica V13.3; Dell; Tulsa, OK, USA).

## Results

### Microglia Activation Early On After Ischemia

**Fig. 2:**
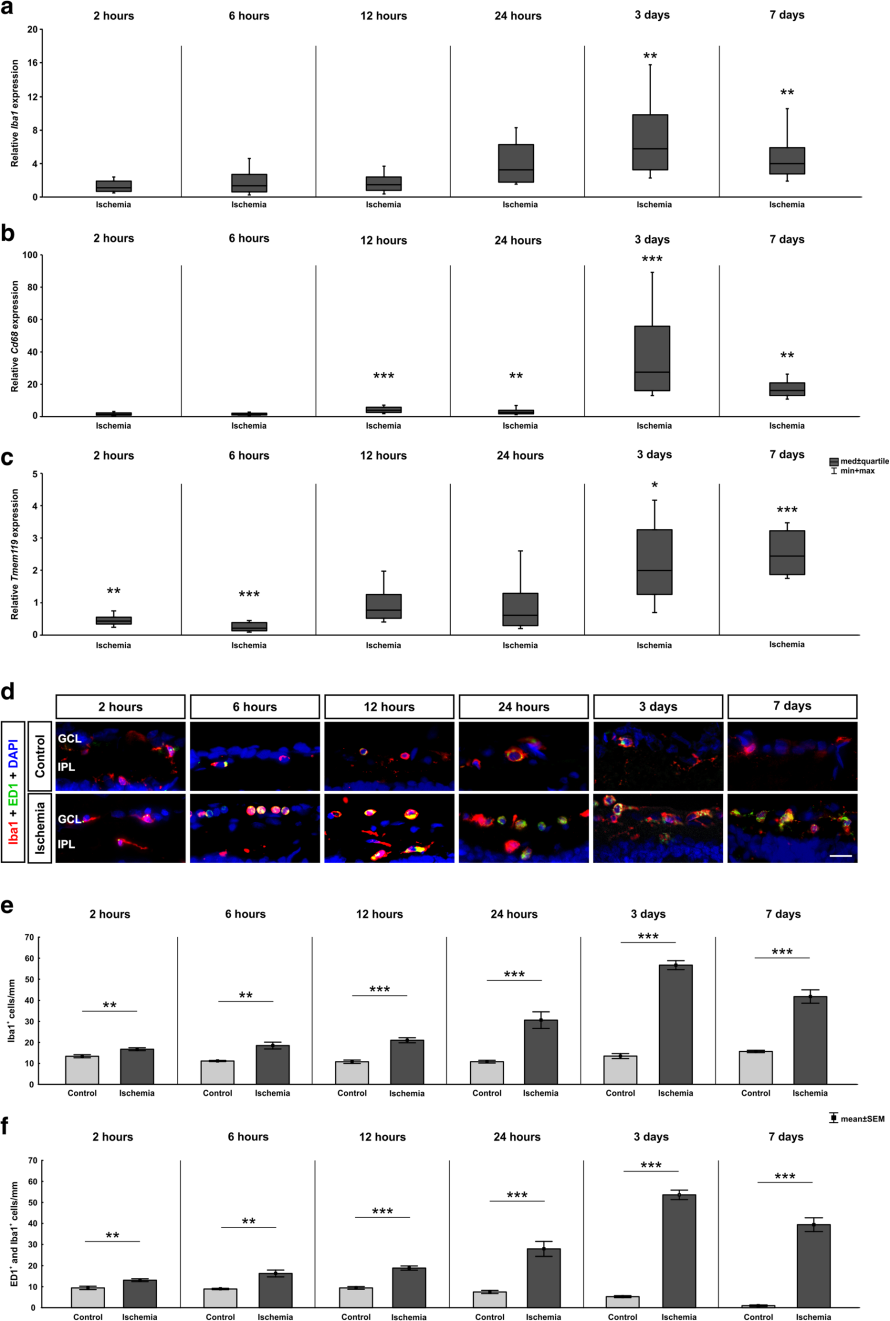
Early microglia/macrophage response after ischemia induction. **a** The relative mRNA expression of the microglia/macrophage marker *Iba1* revealed no differences between ischemic and control eyes after 2, 6, 12, and 24 h. In contrast, after 3 (*p* = 0.001) and 7 days (*p* = 0.001), the *Iba1* mRNA expression level was significantly upregulated in the ischemia group. **b** Additionally, the relative *Cd68* mRNA (activated microglia/macrophages) expression was comparable after 2 and 6 h, whereas after 12 (*p* < 0.001) and 24 h (*p* = 0.008) as well as after 3 (*p* < 0.001) and 7 days (*p* = 0.001) , a significant upregulation was observed in the ischemia group. **c** Analysis of the expression level of the microglia-specific marker *Tmem119* reveled a downregulation 2 (*p* = 0.007) and 6 h (*p* < 0.001) after ischemia induction. In contrast, at 12 and 24 h no differences were detected between both groups. After 3 (*p* = 0.039) and 7 days (*p* < 0.001), a significant upregulation in the *Tmem119* expression was observed in ischemic retinae. **d** Retinal sections were stained with Iba1 (red) to visualize the microglia/macrophage population, while activated ones were detected by an additional staining of ED1 (green). DAPI-labeled cell nuclei (blue). At all time points, microglia/macrophages were present. **e** More Iba1^+^ microglia/macrophages were observed at 2 (*p* = 0.005), 6 (*p* = 0.001), 12 (*p* < 0.001), and 24 h (*p* < 0.001) after ischemia. Also, 3 and 7 days after ischemia induction the microglia/macrophage cell numbers were highly upregulated (both: *p* < 0.001). **f** Significantly more active microglia/macrophages (ED1^+^ and Iba1^+^) were noted in the ischemia group throughout the study. At 2 (*p* = 0.004), 6 (*p* < 0.001), 12 (*p* < 0.001), and 24 h (*p* < 0.001) as well as at 3 (*p* < 0.001) and 7 days (*p* < 0.001), ischemic retinae had higher cell counts. GCL ganglion cell layer, IPL inner plexiform layer. Values are median ± quartile + maximum/minimum for RT-qPCR and mean ± SEM for immunohistology; RT-qPCR: *n* = 5/group; immunohistology: *n* = 8/group. *p < 0.05, **p < 0.01, ***p < 0.001. Scale bar 20 μm

Since it is known that neuroinflammation plays a role in retinal damage following ischemic insult, we analyzed mRNA expression levels of total microglia/macrophages via RT-qPCR. At 2, 6, 12, and 24 h, no alterations in *Iba1* mRNA expression levels were noted in ischemic retinae (*p* > 0.050; Fig. 2a). A significant upregulation of *Iba1* in ischemic animals could be observed at 3 (5.77-fold expression, *p* = 0.001) and 7 days (3.99-fold expression, *p* = 0.001).

The expression levels of *Cd68* (a marker for active microglia/macrophages) were comparable in both groups 2 and 6 h after ischemia (*p* > 0.05; Fig. 2b). At 12 (3.88-fold expression, *p* < 0.001) and 24 h (2.61-fold expression, *p* = 0.008), as well as at 3 (27.49-fold expression, *p* < 0.001) and 7 days (16.15-fold expression, *p* = 0.001), *Cd68* expression was significantly upregulated in the ischemia group.

The relative expression levels of the microglia-specific marker *Tmem119* was downregulated at 2 (0.42-fold expression, *p* = 0.007) and 6 h (0.20-fold expression, *p* < 0.001). In contrast, at 12 (0.77-fold expression, *p* = 0.267) and 24 h (0.60-fold expression, *p* = 0.164), a regulation of *Tmem119* expression was no longer observable. An upregulation of the expression level in the ischemic group was detectable at 3 (1.99-fold expression, *p* = 0.039) and 7 days (2.44-fold expression, *p* < 0.001; Fig. 2c).

Moreover, the total microglia/macrophage population and the ones in an active state were stained for not only 2, 6, 12, and 24 h but also 3 and 7 days after ischemia induction (Fig. 2d). Already at 2 h, ischemic retinae displayed more Iba1^+^ cells then control ones (*p* = 0.005; Table 3; Fig. 2e). These numbers further increased over time (6 h: *p* = 0.001, 12 and 24 h: *p* < 0.001). At 3 and 7 days, microglia/macrophage numbers in the ischemia group had more than doubled compared with controls (both: *p* < 0.001).

**Table 3:** Mean cell counts (±SEM) for cleaved caspase 3, caspase 8, active microglia/macrophages (ED1), total microglia/macrophage population (Iba1), IL-1β, NFkB, TLR3, and TLR4 at all points in time as well as corresponding *p* values. Significant *p* values are marked in italics

	2 h	6 h	12 h	24 h	3 days	7 days
Cleaved caspase 3^+^ cells/mm						
Control	8.07 ± 2.58	0.32 ± 0.10	6.33 ± 0.91	3.61 ± 1.60	0.46 ± 0.23	7.51 ± 2.10
Ischemia	34.51 ± 5.45	15.25 ± 3.07	24.70 ± 2.38	28.05 ± 4.30	5.27 ± 1.14	18.34 ± 3.74
*p* value	*< 0.001*	*< 0.001*	*< 0.001*	*< 0.001*	*0.001*	*0.026*
Caspase 8^+^ cells/mm						
Control	5.86 ± 1.01	3.21 ± 0.37	2.76 ± 0.56	3.71 ± 1.86	4.81 ± 0.47	5.65 ± 0.86
Ischemia	7.40 ± 2.80	14.93 ± 1.40	6.47 ± 0.84	13.71 ± 2.02	4.48 ± 0.86	7.46 ± 1.14
*p* value	*< 0.001*	*< 0.001*	*0.003*	*0.001*	*0.818*	*0.228*
ED1^+^ cells/mm						
Control	9.42 ± 0.80	8.91 ± 0.34	9.42 ± 0.64	7.48 ± 0.75	5.26 ± 0.48	0.95 ± 0.37
Ischemia	13.10 ± 0.69	16.30 ± 1.62	18.88 ± 1.00	27.91 ± 3.56	53.75 ± 2.27	39.41 ± 3.28
*p* value	*0.004*	*< 0.001*	*< 0.001*	*< 0.001*	*< 0.001*	*< 0.001*
Iba1^+^cells/mm						
Control	13.34 ± 0.73	11.08 ± 0.28	10.72 ± 0.81	10.77 ± 0.67	13.46 ± 1.20	15.69 ± 0.57
Ischemia	16.70 ± 0.69	18.44 ± 1.62	20.98 ± 1.19	30.56 ± 3.95	56.76 ± 2.13	41.79 ± 3.13
*p* value	*0.005*	*0.001*	*< 0.001*	*< 0.001*	*< 0.001*	*< 0.001*
IL-1β^+^ cells/mm						
Control	0.32 ± 0.29	0.33 ± 0.44	0.24 ± 0.22	0.28 ± 0.43	0.20 ± 0.34	0.12 ± 0.24
Ischemia	1.87 ± 1.39	1.67 ± 1.07	2.74 ± 2.05	5.28 ± 1.27	6.55 ± 1.91	4.17 ± 1.26
*p* value	*0.008*	*0.005*	*0.004*	*< 0.001*	*< 0.001*	*< 0.001*
NFκB^+^ cells/mm						
Control	9.41 ± 3.13	4.01 ± 1.71	3.81 ± 2.39	5.56 ± 2.76	7.91 ± 2.62	6.76 ± 1.71
Ischemia	18.11 ± 1.89	15.55 ± 2.00	23.64 ± 2.45	22.96 ± 1.02	17.84 ± 1.37	12.45 ± 1.93
*p* value	*0.032*	*< 0.001*	*< 0.001*	*< 0.001*	*0.005*	*0.047*
TLR3^+^ cells/mm						
Control	0.40 ± 0.41	0.41 ± 0.44	0.04 ± 0.11	0.16 ± 0.24	0.04 ± 0.11	0.08 ± 0.16
Ischemia	1.67 ± 1.42	1.35 ± 1.07	2.44 ± 1.72	2.86 ± 1.26	1.98 ± 2.45	0.28 ± 0.31
*p* value	*0.029*	*0.038*	*0.001*	*< 0.001*	*0.042*	*0.128*
TLR4^+^ cells/mm						
Control	5.08 ± 1.19	3.26 ± 0.98	3.46 ± 1.08	2.70 ± 0.63	5.01 ± 1.19	3.55 ± 0.37
Ischemia	9.61 ± 1.26	8.05 ± 0.92	11.84 ± 1.61	10.10 ± 1.25	9.02 ± 0.86	5.55 ± 0.92
*p* value	*0.020*	*0.003*	*< 0.001*	*< 0.001*	*0.016*	*0.067*

Many of these microglia/macrophages were in an active stage (ED1^+^ and Iba1^+^ cells). More active microglia/macrophages were noted 2 (*p* = 0.004), 6 (*p* < 0.001), 12 (*p* < 0.001), and 24 h (*p* < 0.001) after ischemia induction (Table 3; Fig. 2f). These numbers were still highly upregulated at 3 and 7 days (both: *p* < 0.001).

### Early Proinflammatory Cytokine Release After Ischemic Injury

**Fig. 3:**
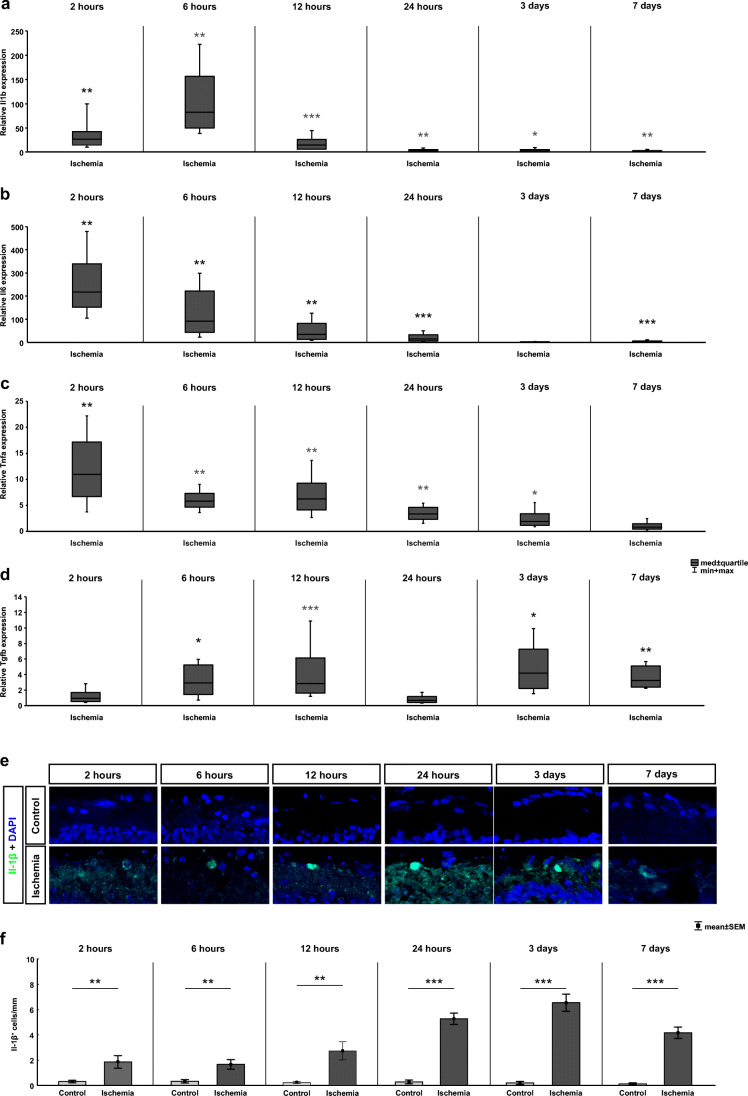
Early inflammatory cytokine alterations after ischemia/reperfusion. **a** RT-qPCR analysis of the *Il1b* expression level revealed an upregulation in the ischemia group at all time points (2 h: *p* = 0.002; 6 h: *p* = 0.006; 12 h: *p* < 0.001; 24 h: *p* = 0.004; 3 days: *p* = 0.031; 7 days: *p* = 0.003). **b** The mRNA expression level of *Il6* was significantly higher in the ischemic group at all early time points (2 h: *p* = 0.006; 6 h: *p* = 0.003; 12 h: *p* = 0.006; 24 h: *p* < 0.001). An upregulation of *Il6* expression was no longer detectable after 3 days, whereas after 7 days, a higher *Il6* expression level was again observed in the ischemia group (*p* > 0.001). **c** The *Tnfa* expression was upregulated at 2 (*p* = 0.004), 6 (*p* = 0.005), 12 (*p* = 0.004), and 24 h (*p* = 0.006) as well as at 3 days (*p* = 0.017). In contrast, a comparable *Tnfa* expression could be noted in both groups at 7 days. **d** Regarding *Tgfb*, its mRNA expression was unaltered at 2 and 24 h but significantly upregulated in the ischemia group at 6 (*p* = 0.022) and 12 h (*p* < 0.001) as well as at 3 (*p* = 0.015) and 7 days (*p* = 0.001). **e** Retinal cross sections were labeled with an anti-IL-1β antibody (green). The cell nuclei were counterstained with DAPI (blue). **f** The number of IL-1β ^+^ cells was significantly higher in ischemic retinae compared with controls at all investigated time points (2 h: *p* = 0.008; 6 h: *p* = 0.038; 12 h: *p* = 0.004; 24 h: *p* < 0.001; 3 days: *p* < 0.001; 7 days: *p* < 0.001). GCL ganglion cell layer, IPL inner plexiform layer. Values are median ± quartile + maximum/minimum for RT-qPCR and mean±SEM for immunohistology; RT-qPCR *n* = 5/group; immunohistology: n=8/group. *p < 0.05, **p < 0.01, ***p < 0.001

The effect of ischemic damage on the expression of the anti-inflammatory cytokines *Il1b*, *Il6*, *Tnfa*, and *Tgfb* was analyzed on mRNA level via RT-qPCR. The expression of all these cytokines was upregulated at an early time point. Already 2 h after ischemia induction, a significant upregulation of *Il1b* mRNA expression was detectable (26.81-fold expression, *p* = 0.002; Fig. 3a). It remained significantly upregulated 6 (82.63-fold expression, *p* = 0.006), 12 (15.02-fold expression, *p* < 0.001), and 24 h (4.02-fold expression, *p* = 0.004) after ischemia. Furthermore, an upregulation of *Il1β* could still be noted at 3 (2.67-fold expression, *p* = 0.031) and 7 days (2.23-fold expression, *p* = 0.003).

The relative gene expression levels of *Il6* were also significantly higher in the ischemic animals in comparison to controls already 2 h after ischemia induction (217.67-fold expression, *p* = 0.006; Fig. 3b). Also, at 6 (91.68-fold expression, *p* = 0.003), 12 (35.30-fold expression, *p* = 0.006), and 24 h (15.16-fold expression, *p* < 0.001), a significantly higher *Il6* mRNA expression was observed in ischemic retinae. At day 3, no significant changes could be seen anymore (1.61-fold expression, *p* = 0.1). However, significantly higher expression levels of *Il6* were again detectable in ischemic retinae after 7 days (4.13-fold expression, *p* < 0.001).

The relative expression levels of *Tnfa* were already upregulated 2 h after ischemia induction (10.95-fold expression, *p* = 0.004; Fig. 3c). Higher expression levels of *Tnfa* were also revealed at 6 (5.80-fold expression, *p* = 0.005), 12 (6.21-fold expression, *p* = 0.004), and 24 h (3.35-fold expression, *p* = 0.006) in ischemic retinae. At day 3, the relative *Tnfa* expression was still higher in the ischemia group than in the control retinae (1.90-fold expression, *p* = 0.017) but was already lower than the expression level measured at 24 h. At 7 days, no difference in the expression level of *Tnfa* was detectable between the ischemic and the control groups (0.82-fold expression, *p* = 0.49).

Additionally, the expression level of *Tgfb* was analyzed via RT-qPCR (Fig. 3d). Interestingly, we did not detect any difference in the relative expression of *Tgfb* in ischemic retinae at 2 (0.93-fold expression, *p* = 0.790) and 24 h (0.68-fold expression, *p* = 0.160), whereas a significant upregulation of *Tgfb* was measured at the early time points 6 (2.92-fold expression, *p* = 0.022) and 12 h (2.84-fold expression, *p* < 0.001). Furthermore, a significant increased expression level was found later, at 3 (4.19-fold expression, *p* = 0.015) and 7 days (3.22-fold expression, *p* = 0.001).

Moreover, an anti-IL-1β antibody was used to label IL-1β in ischemic and control retinae at all investigated time points (Fig. 3e). Significantly more IL-1β^+^ signals were noted in ischemic retinae compared with controls at 2 h (*p* = 0.008), 6 h (*p* = 0.005), 12 h (*p* = 0.004), and 24 h (*p* < 0.001; Table 3; Fig. 3f). In addition, at both late time points, 3 and 7 days after ischemia induction, a high number of IL-1β^+^ signals were noted in ischemia in contrast to controls (3 days: *p* < 0.001, 7 days: *p* < 0.001).

### Toll-Like Receptor Activation During Ischemia

**Fig. 4:**
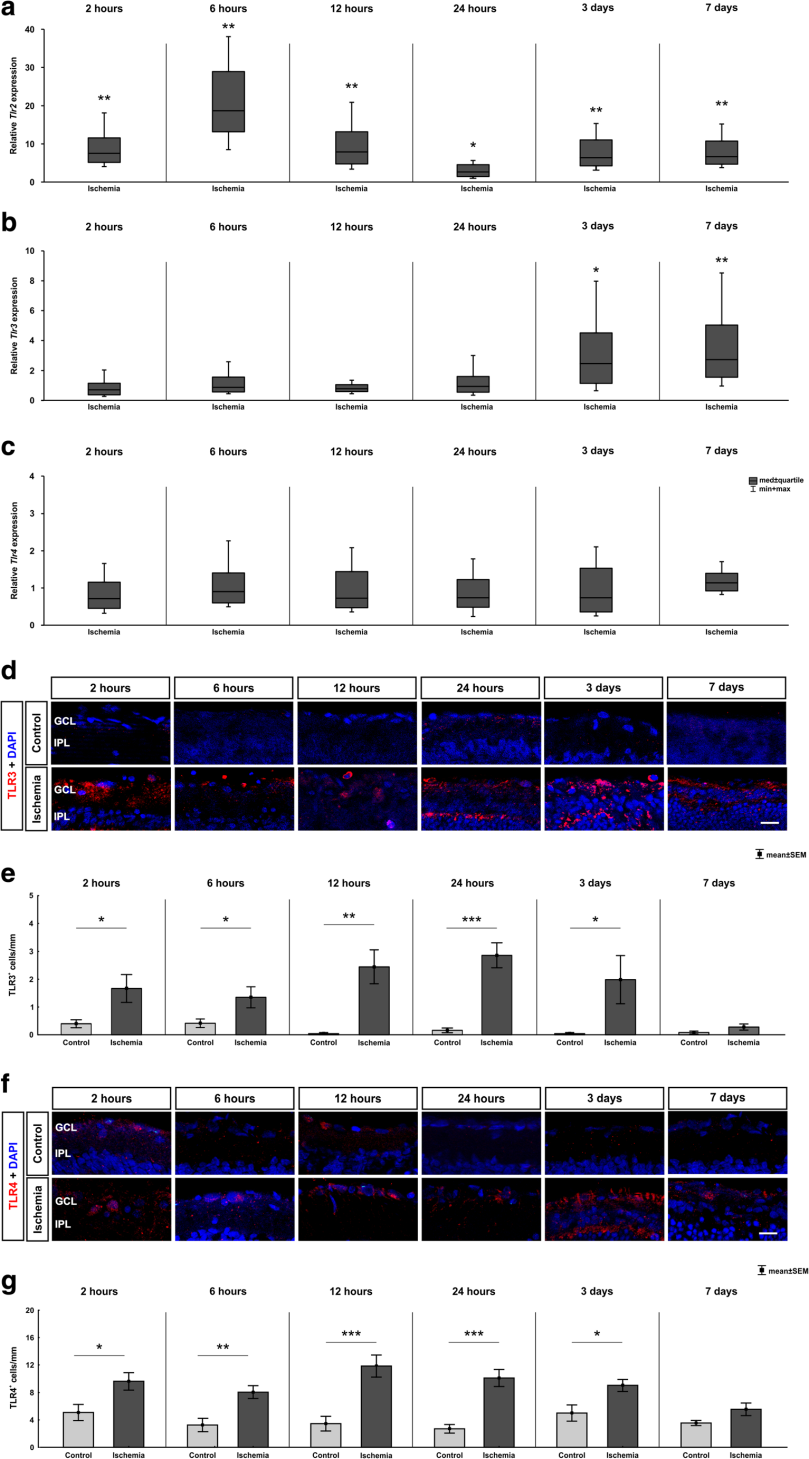
Time-dependent activation of toll-like receptors. **a** On mRNA level, a significant upregulation of *Tlr2* mRNA expression could be detected in the ischemic group at all analyzed time points (2 h: *p* = 0.004; 6 h: *p* = 0.007; 12 h: *p* = 0.004; 24 h: *p* = 0.015; 3 days: *p* = 0.002; 7 days: *p* = 0.003). **b** In contrast, *Tlr3* mRNA expression level was unaltered between 2 and 24 h but was upregulated 3 (*p* = 0.035) and 7 days (*p* = 0.009) after ischemia/reperfusion. **c** No altered *Tlr4* expression could be noted at all time points via RT-qPCR. **d** Exemplary images of retinal cross sections labeled with an anti-TLR3 antibody (red) and cell nuclei were counterstained with DAPI (blue). **e** The evaluation of the amount of TLR3^+^ cells revealed a significant higher number in ischemic retinae compared with control at all time points except 7 days (*p* = 0.128). Already at 2 h after ischemia, a significant difference was noted (2 h: *p* = 0.028; 6 h: *p* = 0.005; 12 h: *p* = 0.004; 24 h: *p* > 0.001; 3 days: *p* = 0.042). **f** Retinal cross sections were labeled with an anti-TLR4 antibody (red) and cell nuclei were counterstained with DAPI (blue). (G) More TLR4^+^ cells were already noted 2 h after ischemia (*p* = 0.020). Counts were still higher at 6 (*p* = 0.003), 12, and 24 h (both: *p* < 0.001). At 3 days (*p* = 0.016), an upregulation was still detectable, but not at 7 days. GCL ganglion cell layer, IPL inner plexiform layer. Values are median ± quartile + maximum/minimum for RT-qPCR and mean ± SEM for immunohistology; RT-qPCR: *n* = 5/group; immunohistology: *n* = 8/group. *p < 0.05, **p < 0.01, ***p < 0.001. Scale bar 20 μm

The activation of cytokines of the innate immune response and inflammatory cell infiltration to damaged areas of the retina could be a result of the stimulation of TLRs by ischemic insults. The relative expression of the *Tlr2* mRNA was upregulated already at 2 h (7.55-fold expression, *p* = 0.004; Fig. 4a). Also, at the later time points, 6 (18.70-fold expression, *p* = 0.007), 12 (7.92-fold expression, *p* = 0.004), and 24 h (2.67-fold expression, *p* = 0.015) as well as at 3 (6.39-fold expression, *p* = 0.002) and 7 days (6.68-fold expression, *p* = 0.003), an upregulation was visible in ischemic retinae.

However, no difference was found in regard to the mRNA expression level of *Tlr3* at 2 (0.72-fold expression, *p* = 0.262), 6 (0.88-fold expression, *p* = 0.607), 12 (0.79-fold expression, *p* = 0.197), and 24 h (0.95-fold expression, *p* = 0.842; Fig. 4b). Interestingly, an upregulation of the *Tlr3* expression was detectable at 3 (2.45-fold expression, *p* = 0.035) and 7 days (2.72-fold expression, *p* = 0.009).

The investigation of the *Tlr4* mRNA expression demonstrated comparable levels in both groups at all investigated time points (2 h: 0.718-fold expression, *p* = 0.160; 6 :h 0.905-fold expression, *p* = 0.643; 12 h: 0.73-fold expression, *p* = 0.262; 24 h: 0.74-fold expression, *p* = 0.301; 3 days: 0.74-fold expression, *p* = 0.399; 7: days 1.14-fold expression, *p* = 0.223; Fig. 4c).

In addition to the RT-qPCR analyzes, immunohistochemical stainings were also carried out with an anti-TLR3 antibody (Fig. 4d). In contrast to mRNA levels, an early significant difference was detected in the number of TLR3^+^ cells in ischemic retina. Already at 2 h, more TLR3^+^ signals were noted in the ischemia group compared with controls (*p* = 0.029; Table 3; Fig. 4e). More TLR3^+^ signals were also present from 6 h up to 3 days (6 h: *p* = 0.038; 12 h: *p* = 0.001, 24 h: *p* < 0.001; 3 days: *p* = 0.042). However, at 7 days, both groups had a comparable number of TLR3^+^ signals (*p* = 0.128).

TLR4 was visualized at all investigated time points using an anti-TLR4 antibody (Fig. 4f). Contrary to RT-qPCR results, a significant higher amount of TLR4^+^ cells was noted in ischemic compared with control retinae 2 h till 3 days after ischemic injury (2 h: *p* = 0.020; 6 h: *p* = 0.003; 12 and 24 h: *p* < 0.001; 3 days: *p* = 0.016; Table 3; Fig. 4g). Seven days after ischemia, we only noted a trend towards an increased number of TLR4^+^ cells (*p* = 0.067).

### Activation of the TLR/MyD88/NFκB Signaling Pathway

**Fig. 5:**
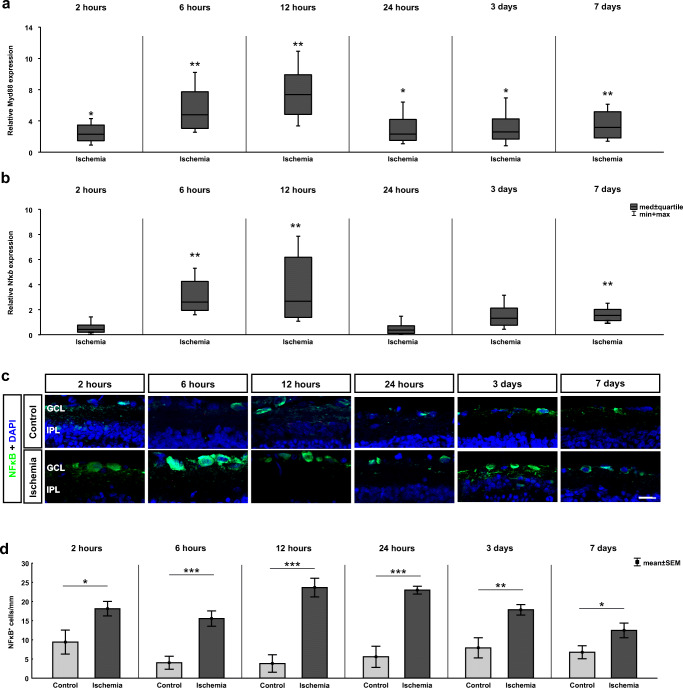
Ischemia-induced TLR downstream effector molecule initiation. **a** The relative gene expression level of the TLR adaptor molecule *MyD88* was significantly upregulated in the ischemia group at all analyzed time points (2 h: *p* = 0.028; 6 h: *p* = 0.001; 12 h: *p* = 0.003; 24 h: *p* = 0.01; 3 days: *p* = 0.024; 7 days: *p* = 0.001). **b** The expression level of the TLR downstream transcription factor *Nfκb* revealed a wave-like shape after ischemia. No changes in the *Nfκb* expression were detectable after 2 and 24 h as well as after 3 days. In contrast, at 6 (*p* = 0.002) and 12 h (*p* = 0.002) as well as at 7 days (*p* = 0.001), a significant upregulation in *Nfκb* expression was detected. **c** Retinal cross sections were labeled with an anti-NFκB antibody (green), and cell nuclei were stained with DAPI (blue). **d** More NFκB^+^ cells were visible in ischemic retinae at all investigated time points (2 h: *p* = 0.032; 6, 12, and 24 h: *p* < 0.001; 3 days: *p* = 0.005; 7 days: *p* = 0.047). GCL ganglion cell layer, IPL inner plexiform layer. Values are median ± quartile + maximum/minimum for RT-qPCR and mean ± SEM for immunohistology; RT-qPCR: *n* = 5/group; immunohistology: *n* = 8/group. *p < 0.05, **p < 0.01, ***p < 0.001. Scale bar 20 μm

The relative gene expression levels of the TRL adaptor molecule *Myeloid differentiation primary response 88* (*Myd88*) were also significantly higher in the ischemic animals in comparison to control retinae at all investigated time points (Fig. 5a). Already 2 h after ischemia induction, a significantly increased *Myd88* mRNA level was noted (2.02-fold expression, *p* = 0.028). Also, at 6 (4.20-fold expression, *p* = 0.001), 12 (6.45-fold expression, *p* = 0.003), and 24 h (2.05-fold expression, *p* = 0.01), a significant upregulation of the *Myd88* mRNA expression was visible in ischemic retinae. At both late time points, 3 (2.28-fold expression, *p* = 0.024) and 7 days (2.79-fold expression, *p* = 0.001), the *Myd88* expression was still higher in the ischemia group.

The ubiquitously expressed transcription factor nuclear factor κB (NFκB), as a downstream regulator of the TLRs signaling cascade, is involved in inflammatory and apoptotic processes (Hayden et al. 2006; Trahtemberg and Mevorach 2017). Therefore, it mediates either cell survival or cell death events. To determine the *Nfκb* expression, specific primers were analyzed via RT-qPCR (Fig. 5b). No change in the *Nfκb* mRNA expression was found between ischemic and control retinae after 2 (0.63-fold expression, *p* = 0.100) and 24 h (0.57-fold expression, *p* = 0.066) as well as at 3 days (1.51-fold expression, *p* = 0.111). Increased expression levels of *Nfκb* were detected at 6 (2.76-fold expression, *p* = 0.002) and 12 h (2.83-fold expression, *p* = 0.002) in ischemic retinae. Moreover, at 7 days, a significant upregulation in the expression level of *Nfκb* was detectable in the ischemic group (1.73-fold expression, *p* = 0.001).

In addition, NFκB was analyzed via immunohistology (Fig. 5c). Already 2 h after ischemia induction, more NFκB^+^ cells were detected in the ischemia group in comparison to controls (*p* = 0.032; Table 3; Fig. 5d). Cell counts were highly increased at 6, 12, and 24 h (all: *p* < 0.001). Even at 3 (*p* = 0.005) and 7 days (*p* = 0.047), more NFκB^+^ cells were seen in ischemic retinae.

### Extrinsic and Intrinsic Caspase–Dependent Apoptotic Pathways

**Fig. 6:**
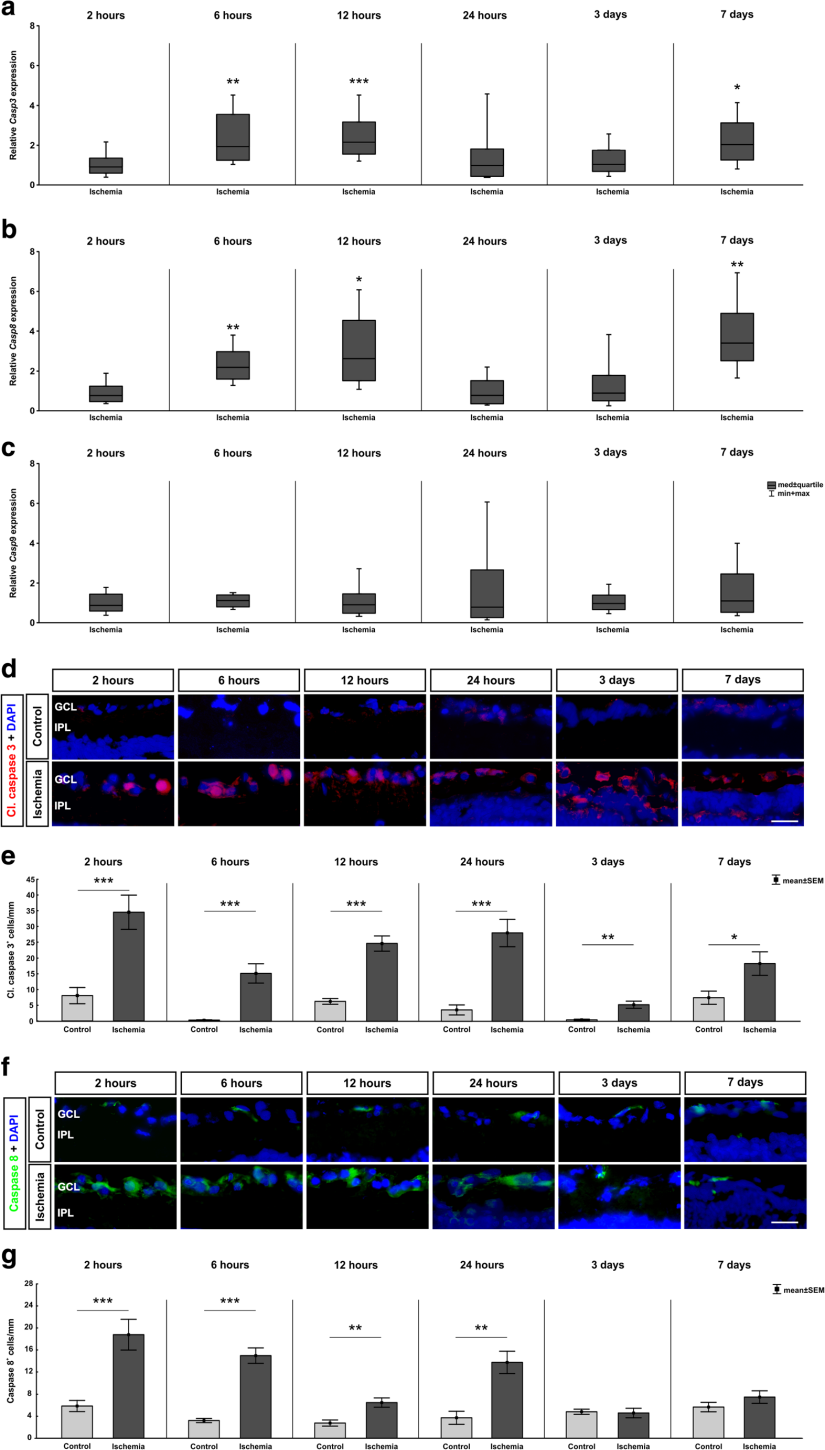
Apoptotic processes after ischemic damage. **a** The relative gene expression level of *Casp3* was significantly upregulated in the ischemia group at 6 (*p* = 0.03) and 12 h (*p* < 0.001) as well as 7 days (*p* = 0.002) after ischemia. Nevertheless, no changes in the *Casp3* expression were visible after 2 and 24 h as well as at 3 days. **b** No changes in the *Casp8* expression were detectable after 2 and 24 h as well as 3 days. In contrast, at 6 (*p* = 0.006) and 12 h (*p* = 0.016) as well as at 7 days (*p* = 0.009) a significant upregulation in *Casp8* expression was discovered. **c** No differences in the relative *Casp9* expression were measured between control and ischemia samples at all investigated time points. **d** Retinal cross sections were labeled with an anti-cleaved caspase 3 antibody (green), while cell nuclei were stained with DAPI (blue). **e** More cleaved caspase 3^+^ cells were seen 2 (*p* < 0.001), 6 (*p* < 0.001), 12 (*p* < 0.001), and 24 h (*p* < 0.001) as well as 3 (*p* = 0.001), and 7 days (*p* = 0.026) after ischemia. **f** Sections were stained with anti-caspase 8 antibody (red). Cell nuclei were visualized with DAPI (blue). **g** More caspase 8^+^ cells were observed in ischemic retinae at 2 h (*p* < 0.001), 6 h (*p* < 0.001), 12 h (*p* = 0.003), and 24 h (*p* = 0.001). At 3 and 7 days, counts in both groups were comparable. GCL ganglion cell layer, IPL inner plexiform layer. Values are median ± quartile + maximum/minimum for RT-qPCR and mean ± SEM for immunohistology; RT-qPCR: *n* = 5/group; immunohistology: *n* = 7/group. *p < 0.05, **p < 0.01, ***p < 0.001. Scale bar 20 μm

Caspase 3 is a member of the cysteine-aspartic acid protease (caspase) family which interacts with caspase 8 and caspase 9*.* The *Casp3* expression was determined via specific primers through RT-qPCR analysis (Fig. 6a). No difference in the *Casp3* expression was observed at 2 (0.91-fold expression, *p* = 0.700) and 24 h (0.98-fold expression, *p* = 0.973) as well as at 3 days (1.04-fold expression, *p* = 0.891). However, a significant upregulated *Casp3* expression was detected at the early time points, namely 6 (1.94-fold expression, *p* = 0.003) and 12 h (2.16-fold expression, *p* < 0.001). At the latest time point of 7 days after ischemia induction, an increase of *Casp3* was also detectable (2.04-fold expression, *p* = 0.021)*.*

The analysis of the *Casp8* mRNA expression also revealed a wave-like upregulation (Fig. 6b). No changes in the *Casp8* mRNA expression were found between ischemic and control retinae at 2 (0.77-fold expression, *p* = 0.315) and 24 h (0.77-fold expression, *p* = 0.442) as well as at 3 days (0.89-fold expression, *p* = 0.703). Enhanced expression levels of *Casp8* were detected at 6 (2.19-fold expression, *p* = 0.006) and 12 h (2.61-fold expression, *p* = 0.016) in ischemic retinae. Also, at 7 days, a significant upregulation in the expression level of *Casp8* was measured in the ischemic compared with the control group (3.40-fold expression, *p* = 0.009). In contrast, the *Casp9* expression revealed no changes at any time points (2 h: 0.87-fold expression, *p* = 0.536; 6 h: 1.12-fold expression, *p* = 0.405; 12 h: 0.91-fold expression, *p* = 0.699; 24 h: 0.78-fold expression, *p* = 0.708; 3 days: 0.97-fold expression, *p* = 0.838; 7 days: 1.10-fold expression, *p* = 0.805; Fig. 6c).

An anti-cleaved caspase 3 antibody was used to visualize apoptosis in retinal cross sections from all time points. Then, cleaved caspase 3^+^ cells in the GCL were counted (Fig. 6d). At 2 h, more cleaved caspase 3^+^ cells were noted in the ischemia group (*p* < 0.001; Table 3; Fig. 6e). This elevation remained throughout the study (6 h: *p* < 0.001; 12 h: *p* < 0.001; 24 h: *p* < 0.001; 3 days: *p* = 0.001; and 7 days: *p* = 0.026).

In addition, caspase 8^+^ cells were labeled with an appropriate anti-caspase 8 antibody (Fig. 6f). Regarding caspase 8^+^ cell counts in the GCL, more cells were observed in ischemic retinae early on in this study (2 h: *p* < 0.001; 6 h: *p* < 0.001; 12 h: *p* = 0.003; 24 h: *p* = 0.001; Table 3; Fig. 6g). This increase was diminished later on after ischemia (3 days: *p* = 0.818; 7 days: *p* = 0.228).

## Discussion

### Time-Dependent Upregulation of Microglia and Macrophages by Retinal Ischemia

Ischemic processes are involved in many CNS diseases and also in retinal ischemia. An activation of microglia and a recruitment of macrophages are noted in numerous neurological disorders. This can be beneficial or detrimental for disease progression (Batchelor et al. 2002; Krady et al. 2005; London et al. 2013; Nakagawa and Chiba 2015; Prinz and Priller 2014). The activation of microglia/macrophages was therefore intensely studied in various retina degeneration models, like glaucoma (O’Koren et al. 2016; Rojas et al. 2014) or ischemia/reperfusion models (Cho et al. 2011; Halder et al. 2013; Santos et al. 2010; Zhang et al. 2005a). However, the role of microglia/macrophages in the response to an ischemic damage in the retina has not yet been clarified. Hence, we analyzed the response of resident and active microglia/macrophages at several time points after ischemic injury. *Iba1* mRNA expression was upregulated at late stages (at 3 and 7 days). Comparing these data to our immunohistochemical analyses, we saw a clear difference. We already noted a significantly higher number of Iba1^+^ cells at 2 h until 7 days after ischemia. Thus, a difference on protein level was  noted earlier than on mRNA level. Nevertheless, we should keep in mind that Iba1^+^ cells were counted in the inner retinal layers (ganglion cell layer and inner plexiform layer), while the mRNA of the whole retina was analyzed. Moreover, this inconsistency could be explained with posttranscriptional and translational regulation.

However, a significant difference in the *Cd68* mRNA expression was observed already 12 h after ischemic damage, which was still present at 7 days. To differentiate between microglia and macrophages, we examined *Tmem119*, which is exclusively expressed in microglia (Bennett et al. 2016; Satoh et al. 2016). Interestingly, the regulation of the *Tmem119* mRNA showed a time-dependent expression pattern. At the early time points (2 and 6 h), we discovered a significant downregulation in ischemic retinae. However, at 12 and 24 h, no differences between the groups were noted. Interestingly, a significantly higher *Tmem119* expression was observed in the ischemia group at the two late time points, 3 and 7 days. This result was surprising, since an activation of microglia was expected due to the retinal injury. Nevertheless, the exact function of Tmem119 in microglia is not known yet (van Wageningen et al. 2019). In brains affected by Alzheimer’s disease, TMEM119 was documented to be expressed on microglia with ramified and amoeboid morphologies (Satoh et al. 2016), indicating a late infiltration by microglia due to time of development of the ischemic damage. The results of van Wageningen et al. showed an unexpected downregulation of the Tmem119 immunoreactivity in white matter lesions of multiple sclerosis patients. They explained this effect with the presence of lymphocytes and inflammatory mediators (van Wageningen et al. 2019). The inflammatory recruitment of microglia/macrophages seems to need time to respond to the ischemic damage.

### Early Cytokine Release

In this study, we analyzed the mRNA expression of various cytokines, like *Il1b*, *Il6*, *Tnfα*, and *Tgfβ*. Regarding *Il1b*, *Il6*, and *Tnfα*, they already reacted 2 h after ischemia with an elevated expression. This upregulation was constant during all investigated time points for *Il1b*. This could also be demonstrated on protein level using an anti-IL-1β antibody. *Il6*, on the other hand, was not upregulated at 3 days and T*nfα* was not higher expressed at 7 days. Hangai et al. identified RGCs, endothelial cells, and recruited neutrophils in the retina as the source of *Il1b* (Hangai et al. 1995). They suggested that IL-1β plays an essential role in ischemic injuries. In their ischemia rat model, they induced the damage via a ligation of the optic nerve. Three hours after the induction, they documented an early upregulation in the *Il1b* expression with two peaks, 12 and 96 h after ischemia (Hangai et al. 1996). The analyzed cytokines *Il1b*, *Il6*, *Tnfa*, and *Tgfb* all play important roles in various neurological disorders and have multiple functions involving cell differentiation, growth, and survival (Gruol and Nelson 1997; Yafai et al. 2014). Interestingly, previous studies demonstrated a neuroprotective effect on inner retinal neurons including RGCs after retinal ischemia (Loddick et al. 1998; Sanchez et al. 2003). Sanchez et al. confirmed also that microglial/phagocytic cells are the major source of retinal IL-6 after an injury (Sanchez et al. 2003).

A deleterious role after ischemia/reperfusion injury was postulated for TNFα (Berger et al. 2008). Berger et al. noted that a neutralization of TNFα improved the retinal function in an in vivo model based on p55 and p75 knockout mice and rats with an elevated IOP of 120 mmHg for different durations from 30 to 60 min (Berger et al. 2008). This demonstrated a pivotal role of TNFα in the immune response to an ischemic damage. Striking was the wave-like expression of *Tgfb* in our study. An elevated mRNA level was only notable at 6 and 12 h and at the late time points, 3 and 7 days after ischemia. However, at 2 and 24 h, no significant difference was visible between the groups.

### Early TLR2 and TLR3 Activation in Ischemic Retinae

Next, we investigated different members of the evolutionarily conserved TLR family, which can be divided into different subfamilies based on their individual recognition pattern (Akira and Takeda 2004; Beutler 2004; Janeway and Medzhitov 2002). Whereas TLR2 recognizes lipids and peptidoglycans, TLR4 recognizes different ligands like lipopolysaccharides, fibronectin, and heat shock proteins (Akira et al. 2006; Kielian 2006). In human glaucomatous retinae, TLR2, TLR3, and TLR4 were overexpressed. Thereby, TLR2 and TLR4 were distinctively noted in microglia cells, while TLR3 expression was prominently seen in astrocytes (Luo et al. 2010). TLR2 and TLR4 are located on the cell surface, while TLR3 is found solely in intracellular compartments. Adaptor proteins, such as MyD88, are essential for the mediation of the TLR downstream signaling pathway, except for TLR3 (Akira and Takeda 2004).

TLRs respond to pathogens, multiple cytokines, and environmental stress through a quick modulation. We noted a significant increased *Tlr2* mRNA expression starting at 2 h until 7 days. Interestingly, we found a significant upregulation of the *Tlr3* expression only at the both late time points (3 and 7 days). In addition, we noted a different regulation on protein level via TLR3 staining. A significantly increased number of TLR3^+^ cells was revealed at all investigated time points in ischemic compared with control retinae, except at the latest time point of 7 days. In contrast, no difference in the *Tlr4* expression pattern was detected throughout the study. These results suggest that the immune response to the retinal damage is mediated via TLR2 starting at 2 h. In addition, the Myd88-independent pathway via TLR3 seems to be activated. TLR4 and the associated downstream pathway on the other hand seem to be not exclusive in the immune response to retinal damage.

TLR2 is one of the most intensely investigated members of the TLRs relating to neurodegenerative disorders (Guillot-Sestier and Town 2018). However, previous studies mainly focused on TLR4 after ischemic injury as it is known that necrotic cells release endogenous ligands, which can be recognized by TLRs and activating an innate immune response (Dvoriantchikova et al. 2010a; Dvoriantchikova et al. 2010b; Fujita et al. 2009; Piccinini and Midwood 2010). It is worth noting that there seems to be a certain contradiction to the results of other groups, who also examined the role of TLR2 and TLR4 in different ischemia/reperfusion models (Dvoriantchikova et al. 2010b; Ishizuka et al. 2013; Qi et al. 2014). Qi et al. found a significant upregulation of *Tlr4* starting 1 h after injury, while they could not find an altered *Tlr2* expression (Qi et al. 2014). Therefore, they suggested that the TLR4 signaling pathway activates the NLRP3 inflammasomes. Nevertheless, their results were obtained from a different ischemia model. Our model is based on IOP-raising, while Qi et al. generated damage by ligating the central retinal artery (Qi et al. 2014). Ishizuka et al. used a retinal ischemia mouse model, where they ligated the arteries. Via Western blot analysis and immunohistochemistry, they observed more TLR4 1 and 19 h after ischemia. Additionally, they noted reduced damage after ischemic injury in TLR4 knockout mice (Ishizuka et al. 2013). The TLR4 staining of our study supports these findings, as we noted an upregulation of TLR4 starting at 2 h until 3 days after ischemia. Comparable findings of a TLR2 dominance over TLR4 under stress conditions like ischemia/reperfusion damage were also seen by Bagchi et al. in cardiac tissue of wild-type and IL-10^−/−^ mice and rats (Bagchi et al. 2017). They induced the ischemia via cannulation of the aorta. Interestingly, they measured a differential expression of TLR2 and TLR4 and proved a TLR2 increase contrary to TLR4 in IL-10^−^/^−^ mice, indicating a more prominent role of TLR2 in the immune response under ischemic conditions which needs to be analyzed further.

### MyD88 Predominantly Activates Wave-Like NFκB-Mediated Response

As mentioned before, Myd88 is an adaptor molecule, which is critical to mediate the induction of inflammatory cytokines via NFκB stimulated by TLR pathways (Sugiyama et al. 2016). The results of our study indicate an activation of Myd88 via TLR2 signaling. This activation takes place simultaneously with the upregulation of TLR2. Thus, we noted a higher *Myd88* mRNA expression from 2 h to 7 days.

An activation of the transcription factor NFκB was documented after ischemic injury in the CNS (Chen et al. 2003; Ridder and Schwaninger 2009; Zeng et al. 2008; Zhang et al. 2005b). NFκB, a key regulator of cell survival and inflammatory responses, is expressed by neuronal and glial cells (Oeckinghaus and Ghosh 2009). We observed time-dependent changes regarding *Nfκb* mRNA levels after ischemic injury. At the early time points, a significant upregulation was followed by a gradual decrease. At 3 days, *Nfκb* mRNA levels in both groups were comparable, while we observed another distinct increase at 7 days. Nevertheless, we did not note an early upregulation of *Nfκb* mRNA in contrast to Wang et al. (2006). They also used an ischemia/reperfusion model with high IOP, but only applied 110 mmHg. However, we observed a wave-like decrease from 6 to 24 h, reaching a peak at 12 h. Wang et al. found comparable *Nfκb* mRNA levels, though they claimed to see the peak at 4 h after ischemia, followed by gradual decrease towards 24 h (Wang et al. 2006).

### Enhanced Caspase Activation

It is known that RGCs are the most sensitive cell type to ischemic injury. Sellés-Navarro et al. induced ischemia over several periods, from 30 to 120 min, by increasing the IOP in rat eyes. They performed examinations at 5, 7, 14, and 30 days after injury to investigate the survival of RGCs (Selles-Navarro et al. 1996). They noted a loss of RGCs already after 5 days. In a previous study by our group, we also noted that RGCs are in particularly damaged after the ischemic insult, with a progressive degeneration (Palmhof et al. 2019b).

Apoptosis via activation of caspases leads to rapid cell death. Caspase 3 is one member of the caspase execution subfamily (Degterev et al. 2003). In our study, significantly more cleaved caspase 3^+^ cells were noted in the GCL of ischemic retinae at all investigated time points. Also, mRNA levels of *Casp3* were found to be upregulated in early and late stages. Yang et al. observed more caspase 3 protein 6 h after ischemia in rats (Yang et al. 2011). Also in other organs, like in the kidney, ischemia-induced injury activates apoptosis through caspase 3 activity (Meng et al. 2020).

TLR4 can lead to an increased caspase 8 expression in a retinal ischemia-reperfusion model (Chi et al. 2014), which is in accordance with our study results. The authors postulate that a subsequent production of IL-1β occurs. Caspase 8 activation can also be engaged by inflammasome components. Inflammasomes are cytosolic complex that process cytokines, like IL-1β, in their active forms (Tummers and Green 2017).We noted a higher *Il1b* mRNA expression in ischemic retinae as well as more IL-1β^+^ cells throughout the study. Knockout of TLR4 signaling suppressed caspase 8 activation and RGC death (Chi et al. 2014). Shabanzadeh et al. observed that caspase 8 inhibition decreases the neuropathological consequences of cerebral or retinal infarction in a retinal stroke model (Shabanzadeh et al. 2015).

## Conclusion

**Fig. 7:**
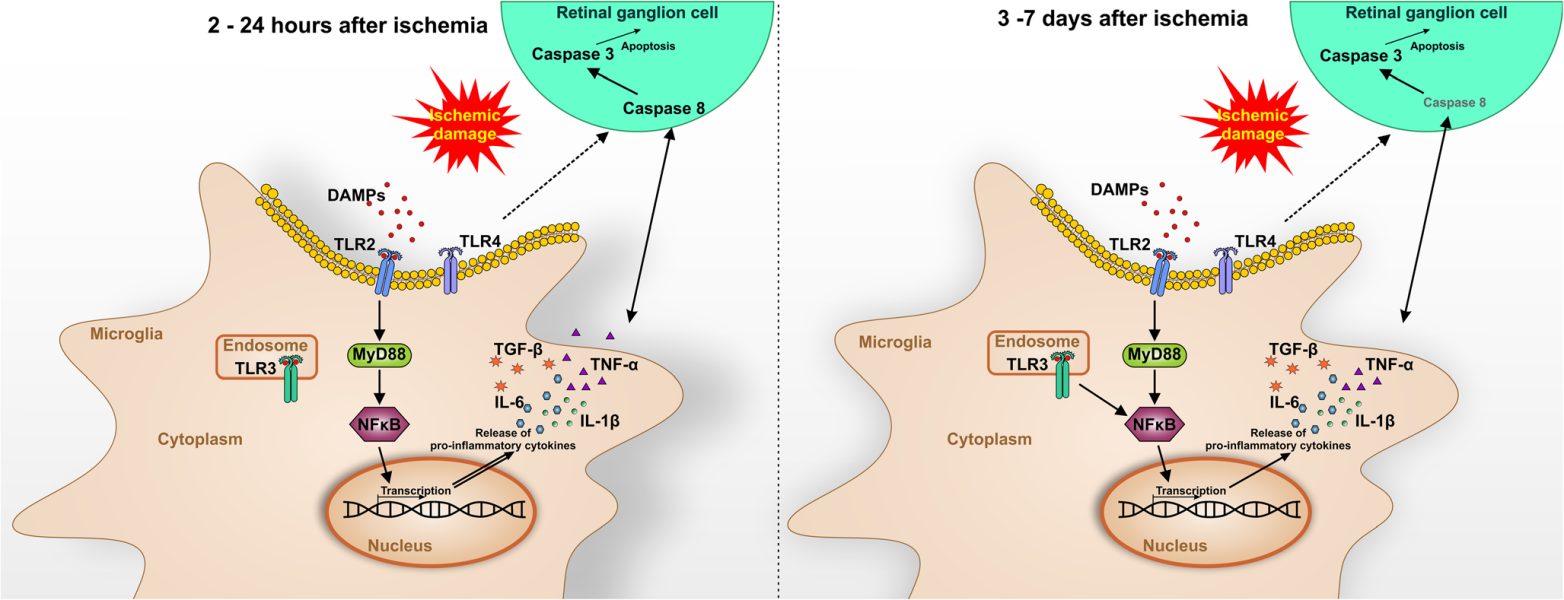
Schematic overview of study results. Retinal ischemia/reperfusion led to a progressive microglia activation. At early time points (2 to 24 h) after an ischemic injury, the immune response seemed to be mediated mainly by TLR2. The following cascade included MyD88, which activated NFκB. This transcription factor led to the release of proinflammatory cytokines, such as IL-1β, IL-6, TNFα, and TGFβ. TLR3 was also upregulated and a MyD88-independent signaling pathway was activated in the immune response via NFκB. An activation of the initiator caspase 8 in retinal ganglion cells was noted at early time points, while the effector caspase 3 was enhanced throughout the study

In summary, we could demonstrate a strong, progressive activation of microglia/macrophages throughout this study. Cytokine regulations were already noted a few hours after ischemia and continued until 7 days. Furthermore, the damage was associated with an early activation of TLR2, TLR3, and their downstream signaling cascade. These findings were accompanied by apoptotic mechanisms throughout the study (Fig. 7).

## Electronic Supplementary Material

Supplemental Figure 1: Exemplary H&E stained retinal cross-sections of all points in time from 2 hours until 7 days after ischemic damage. A reduction of the total retinal thickness was observed in the ischemia group at 3 and 7 days. GCL ganglion cell layer; IPL inner plexiform layer; INL inner nuclear layer; OPL outer plexiform layer; ONL outer nuclear layer. Scale bar: 20 μm. (PNG 6630 kb)

High Resolution (TIF 9691 kb)

## Data Availability

Data is included in the manuscript.
