# Supplementary figures and images for: Microglia Activation in Retinal Ischemia Triggers Cytokine and Toll-Like Receptor Response
Source: J Mol Neurosci. 2020 Aug 24;71(3):527–44. doi: 10.1007/s12031-020-01674-w (PMC8575759; doi:10.1007/s12031-020-01674-w)

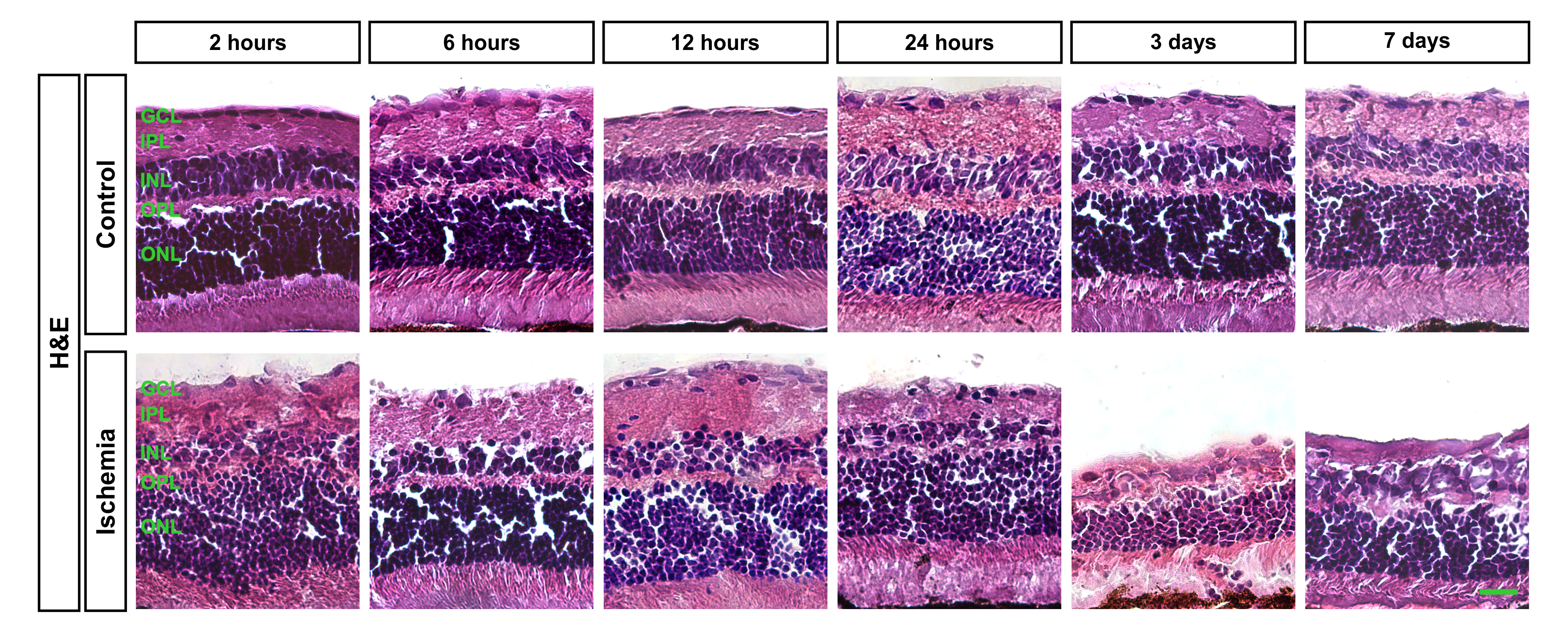

Supplement: Supplementary file 1 — Exemplary H&E stained retinal cross-sections of all points in time from 2 hours until 7 days after ischemic damage. A reduction of the total retinal thickness was observed in the ischemia group at 3 and 7 days. GCL ganglion cell layer; IPL inner plexiform layer; INL inner nuclear layer; OPL outer plexiform layer; ONL outer nuclear layer. Scale bar: 20 μm. (PNG 6630 kb) [file 12031_2020_1674_Fig8_ESM.png]

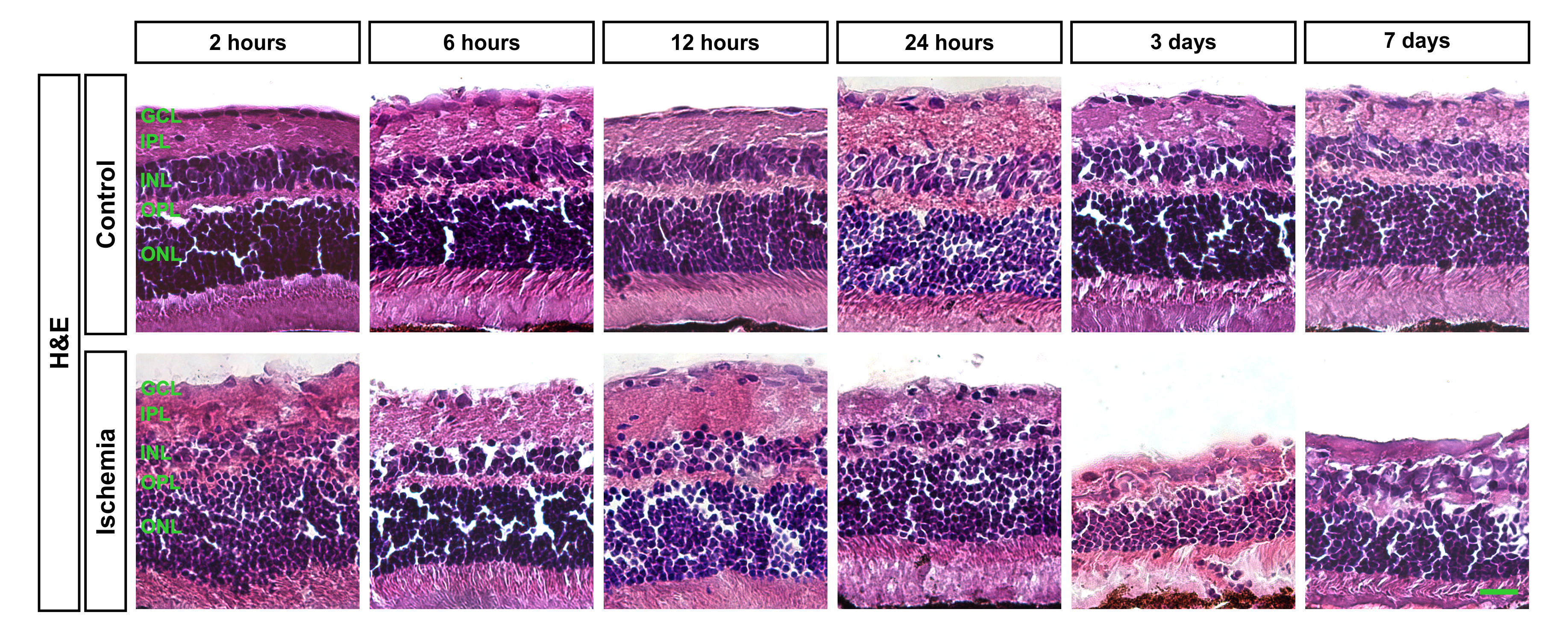

Supplement: Supplementary file 2 — High Resolution (TIF 9691 kb) [file 12031_2020_1674_MOESM1_ESM.tif]
